# Eating disorders and palliative care specialists require definitional consensus and clinical guidance regarding terminal anorexia nervosa: addressing concerns and moving forward

**DOI:** 10.1186/s40337-022-00659-x

**Published:** 2022-09-06

**Authors:** Joel Yager, Jennifer L. Gaudiani, Jonathan Treem

**Affiliations:** 1 Department of Psychiatry, University of Colorado School of Medicine, Aurora, CO USA; 2 Gaudiani Clinic, Denver, CO USA; 3 Department of General Internal Medicine, University of Colorado School of Medicine, Aurora, CO USA

## Abstract

**Background and objectives:**

Premature deaths are estimated to occur in 5–20% of patients with anorexia nervosa (AN). Among them, some patients with severe and enduring anorexia nervosa (SE-AN) will die due to the medical complications of malnutrition or to suicide. Almost no literature provides guidance to patients, clinicians, and loved ones regarding clinical characteristics of those with SE-AN who recognize and accept the fact that they will not be able to survive their disease. Consistent with general medical literature on terminal illness and based on the authors’ work with patients at this phase of life, we previously described four clinical characteristics of the small group of SE-AN patients who may be considered to have a terminal eating disorder. Following publication of this article, several opinions objecting to these formulations were published. The goals of this article are to respond to the key themes of concern posed by these objections, to extend our discussion of the palliative care and associated needs of these patients and their families, and to suggest ways in which the eating disorder and palliative care fields might develop more definitive criteria and consensus guidelines for the assessment and management of these patients.

**Methods:**

Based on a selective narrative review of the literature, our combined experiences with these patients, and clinical reasoning, we address critiques grouped around five major themes: that (1) labels such as terminal AN are dangerous; (2) since AN is a treatable disorder, no SE-AN patients should be considered terminal; (3) a terminal psychiatric condition cannot be defined; (4) the proposed definition is not specific enough; and (5) considerations regarding mental capacity in the proposed criteria do not sufficiently account for the psycho-cognitive impairments in AN.

**Results:**

Our analysis responds to the critiques of our original proposed clinical characteristics of those with terminal AN. While refuting many of these critiques, we also appreciate the opportunity to refine our discussion of this complex topic and identify that there are multiple stages of SE-AN that can result in good clinical outcomes. Only when all of these have failed to provide adequate amelioration of suffering do a low number of patients progress to terminal AN.

**Conclusions:**

By further refining our discussion of terminal AN, we aim to encourage eating disorders and palliative care specialists to develop expert consensus definitions for terminal AN and to generate authoritative clinical guidance for management of this population. By validating terminal AN as a distinct condition, patients with this subcategory of SE-AN, their families, and their caregivers facing end-of-life concerns may be better able to access palliative and hospice care and related services to help improve their overall experiences at this phase of life.

## Introduction

A recently published manuscript by Gaudiani et al. sought to provide a clinical framework to define “terminal anorexia nervosa (AN).” That framework acknowledges the existence of and right to appropriate care for a select, rare group of adults with severe and enduring anorexia nervosa (SE-AN) who choose to stop engaging in life-prolonging care with the understanding that death will ensue. It attempts to begin a conversation in the eating disorder and palliative care fields that many have deemed long overdue, by describing clinical criteria these patients held in common, which might be considered by clinicians caring for other patients requesting similar goals of care.

Based on sources in the eating disorders literature noted below, prior literature on criteria for clinical terminality [1, 2], and our clinical experiences [3, 4], our proposed criteria for *terminal AN* [3] were as follows: (1) diagnosis of AN, (2) age over 30, (3) prior persistent engagement in high-quality multidisciplinary eating disorder care, and (4) while demonstrating decision making capacity to decide that further treatment will be futile, deciding not to engage in treatment that would prolong life and accepting death.

Since our article appeared, several criticisms of both the criteria and the concept of terminal AN have been published [5, 6]. Despite these critical misgivings, the facts remain that unrelenting AN has a high mortality rate and that eating disorder specialists and palliative care specialists remain underprepared to guide end-of-life care in this population. The goals of this article are to respond to the key themes of concern posed by these objections, to extend our discussion of the palliative care and associated needs of these patients and their families, and to suggest ways in which the eating disorder and palliative care fields might develop more definitive criteria and consensus guidelines for the assessment and management of patients with end-stage SE-AN.

Importantly, the aim of these proposed criteria is to offer validation, legitimacy, and clinical guidance for adults who choose comfort-focused care and who meet all the criteria. Without this protection, such patients face indefinite ongoing pressure to retry treatment modalities that have proven unhelpful and harmful, with potential associated loss of autonomy (at times through the legal system). Their loved ones also are denied permission to shift their own role from medical custodian characterized by discord and substituted judgement to therapeutic advocate characterized by loving alignment, compassion, and acceptance.

For background, despite rigorous and exhaustive efforts in the field of anorexia care, a consistent subset of patients dies of complications of their illness. Premature deaths are estimated to occur in 5–20% of patients with AN [7], with this broad range reflecting differences in the specific patient population studied. Among them, patients with SE-AN have higher mortality risks. [8, 9]. A prospective cohort of 384 patients with severe AN requiring inpatient hospitalization had a standardized mortality ratio (SMR) for women of 15.9 and for men 22.4. Older age predicted mortality, with mean age at death 41.3 (± 15.3) years; those admitted between ages 30–34 had the highest SMR of 26. Somatic (medical) causes accounted for 43% of deaths, with 11.5% of deaths attributed to suicide [8]. Individuals with AN are 18 times more likely to die by suicide [10].

A subset of patients with SE-AN are repeatedly subjected to higher level of care treatments that have previously proven futile in their care and that some patients experience as traumatizing and intolerable. At various points in their illness trajectories, some patients ultimately decide that they have suffered too long and too deeply. They may no longer want to subject themselves to further active treatments inevitably followed by prompt relapses and ongoing anguish, and even acknowledge that refusing further intensive treatments means they will die. These patients often find that their providers are uncomfortable with or underprepared to discuss end-of-life care options. Providers who do wish to engage these conversations are hampered by a paucity of literature to guide their recommendations. This often results in provider responses that coerce or compel patients to continue with standard disease-directed therapies. By contrast, patients with metastatic cancer are unlikely to be compelled to repeat treatments that have previously failed to halt progression of their illnesses. Rather, they are more likely to receive the psychological preparation, connection, and medical and emotional support offered via palliative and hospice care to patients with terminal conditions.

As investigators critical of defining a terminal stage of anorexia have pointed out, bio-prognostic factors in SE-AN have yielded imprecise results. We therefore must rely on close clinical observations and meaningful narratives of patients to characterize terminal phases of SE-AN [11–13]. Validating the existence (and rights) of those SE-AN patients facing death is vital based on principles of body autonomy, patient choice including in the setting of chronic mental illness, and the fundamentals of *primum non nocere*.

To be clear, we see SE-AN and the terminal phase of AN as distinct conditions. Most importantly, SE-AN is a chronic condition of undetermined duration and course. In contrast, the much less common stage of terminal AN, which most often occurs during a late phase of SE-AN, is a time-limited condition marked by progressive deterioration and fatal outcome. Given that eating disorders are variably ego-syntonic, that malnutrition can alter cognitive function, and that much eating disorder recovery work may involve assertions of power by clinicians and loved ones over patients in order to break the eating disorder’s hold, we must be precise in attempts to delineate the small population of patients we regard as terminal, whose management stands outside of standard care protocols for AN. This delineation is based exclusively on the *patient’s* self-determination and choice not to engage in life-saving measures anymore, once they have received every reasonable opportunity to recover. We hold that a small group of patients may ethically decline care measures they no longer feel will be helpful, knowing that a subset of these patients will go on to die of the medical complications of malnutrition while designated as having terminal AN, and that an even smaller subset of these patients may choose to use medical aid in dying (MAID) medications in jurisdictions where such options are legal.

## Methods

Methodology, rationale, and explication of proposed criteria, described above, for ‘terminal anorexia’ were delineated in Gaudiani et al. [3]. To supplement our clinical observations, a PubMed selective narrative literature search was conducted using the following terms in article’s titles “anorexia nervosa” AND the following additional terms individually “mortality” (46 articles),“death” (34 titles); palliative care (5 titles), “MAID”, “medical aid in dying” (zero titles); assisted suicide” (zero titles) and end of life (one title). Via snowballing, for each article from these lists that were relevant to the aims of this article, PubMed titles listing “similar articles” and “cited by” articles were also examined. We used both forward and reverse snowballing, examining pertinent articles' references and searching for more recent articles citing the original article after publication. As initial strategies, these procedures have been shown to be good alternatives to the use of database searches [14, 15].

Since publication of our article, to identify specific concerns about our proposals, a supplemental literature review was conducted through PubMed to collect relevant response articles in the subsequent literature. Two titles met inclusion criteria: Guarda et al. [5] and Riddle et al. [6]. Additional concerns were derived from comments provided by anonymous reviewers in response to our submitted manuscripts.

## Results

Based on these two articles [5, 6] and anonymous review comments, we identified critical responses or concerns (presented in italics) that can be grouped into five categories, each of which we address separately. Our analysis reveals that the critiques of our presentations all rest on substantially questionable premises.

### Concern 1: Labels are dangerous

Concern is expressed for vulnerable patients and their caregivers who would find that terminology demoralizing, embedded with the supposition that additional disease-directed treatment would not benefit them. Guarda et al. propose that over-identification with the term ‘terminal’ may precipitate social contagion of suicide, and increased demand for medical aid in dying (MAID) among this group. The argument presented here is that the term ‘terminal’ carries an implication that may precipitate a self-fulfilling prophecy.

## Discussion

As we have tried to demonstrate in the introduction, patients die of anorexia and its complications despite maximal therapies. Rejecting the notion of “terminal AN” does not change the mortality rate or clinical reality that some patients will choose to seek compassionate support at the end of their lives. Being able to accurately identify a population who may reasonably be expected to die of an illness is a way of encouraging vital conversations between patients and their providers, including conversations that may portend more intensive treatments and eventual recovery. It is a way to align a care team and patient on what is critically important as the gravity of illness deepens. It does not precipitate a terminal event any more than labeling a cancerous condition as terminal causes the cancer to grow. Rather, this acknowledgement allows a richer, more nuanced conversation around patients’ goals and values. Further, to deny patients the validation that their condition may proceed to death is to create a therapeutic rift that may itself be demoralizing.

Guarda et al. express forebodings around social contagion of suicide and ‘concierge’ practitioners unscrupulously providing aid-in-dying services to anorexia patients but advance no evidence to support these concerns. This argument is a type of doomsaying meant to engender apprehension in the context of uncertainty, and without supporting evidence, likely doesn’t have a place in academic discussion.

Guarda et al. note that increased public demand for MAID in psychiatric illness in Europe following legalization is a ‘warning’. MAID refers to the prescription of medication for a terminally ill, mentally capable adult with a prognosis of six months or less to live, which the patient may self-ingest to choose the time and manner of a death that will otherwise occur due to the primary disease process. While groups across the world for personal, religious, or cultural reasons may reject the idea of MAID and fear a theoretical expanded or nefarious use, it has been legalized so far in 8 US states and 11 countries. Indeed, Canada has approved legislation to take effect in 2023 that will allow individuals with “irremediable psychiatric suffering” to seek MAID, regardless of an otherwise terminal medical condition [16].

It would be useful to acknowledge that in context, psychiatric patients who participate in MAID are still greatly outnumbered by the number of psychiatric patients who die by suicide. For example, in the Netherlands from 2011 to 2014, a total of 110 psychiatric patients were reported to have received MAID, of whom only 4 women (3% of series) had eating disorders with borderline personality disorder [17]. Although more recent data reports from Belgium do not break out the eating disorder diagnosis specifically, the number of psychiatric patients requesting assessment for MAIDs remains notably low. In 2020, out of 2444 requests for MAID evaluations in Belgium, only 21 (0.9%) were for psychiatric disorders, which included a wide range of personality, mood, PTSD and autism spectrum disorders, in addition to possible eating disorder [18]. Similarly, in the Netherlands in 2020, among 7,459 reports of termination of life on request, only 115 (1.5%) were for the entire gamut of psychiatric conditions [19]. Furthermore, the implied rise in MAID requests for psychiatric conditions in Europe may be better characterized as evidence that there existed a degree of intractable suffering which now has recourse.

### Concern 2: Anorexia nervosa is a treatable disorder [5, 6]

This critical appraisal rightly asserts that clinicians who treat AN cannot predict who will recover or when, nor identify “those who will not be able to survive”, as most medical complications are reversible with nutritional rehabilitation and expert care. Therefore, one cannot safely identify terminality in those with AN.

#### Discussion

The premise of this argument is a propositional fallacy that presupposes treatable conditions cannot be terminal. In fact, terminal conditions are not mutually exclusive with treatable conditions. To take an example from somatic medicine, consider renal failure. Anuria proceeds to death within days to short weeks. However, dialysis meaningfully extends life on the order of years to decades. Should a patient in renal failure with capacity refuse dialysis for personal, cultural, value-based, or spiritual reasons, this treatable condition becomes terminal. The same is true in SE-AN. Should a patient affirm clearly, consistently, in accordance with their values, and in acceptance that the outcome of their decision is death, that they will not pursue disease-directed therapies, starvation leads to death and is itself a terminal condition. This is the rationale behind the fourth clinical characteristic necessary for this condition to be considered terminal: “Consistent, clear expression by an individual who possesses decision-making capacity that they understand further treatment to be futile, they choose to stop trying to prolong their lives, and they accept that death will be the natural outcome.” In our formulation, individuals not meeting this criterion are disqualified from being considered terminal. Key to this concept is the acceptance by clinicians and families that through a rigorous evaluation of capacity (see below), a patient with SE-AN who possesses capacity has the ability to define what type of medical care they will or will not accept in accordance with ethical principles of autonomy. Patients with capacity who choose an avenue of medical care contrary to the values of their treating practitioners should still be able to make that choice. In some ways, this is analogous to patients with alcohol use disorder who refuse to stop drinking despite being aware that they may die of the disease. As a culture and a profession, we tend to assert that suffering that extends life is ethically permissible and palliation that shortens life is ethically tenuous. For that reason, it can be difficult for providers to align with patients who conclude that further attempts at treatment will simply prolong their agony without meaning or purpose.

Guarda et al. describe their experience with patients with severe AN, noting: “when a patient’s life is at risk, involuntary treatment provided by a behavioral inpatient specialty program can be lifesaving, and when effective is often met with gratitude by patients.” Inherent in this position is a form of positive outcomes bias, where providers remember the gratitude of the patients whose conditions they were able to reverse, but fail to account for their role in the suffering of patients who died despite involuntary treatment. In exclusive recognition of saved patients, Guarda et al. disregard what is done to those who were not saved, often against their will. There is evidence in the literature to support this. For example, in a series of 109 patients placed on involuntary legal certification in an eating disorder program, 24% of the certifications were terminated as the treatment was not found to be helpful [13]. Some patients report that involuntary hospitalizations can be traumatizing. Van Elburg et al. [20] state,”CT [compulsory treatment] is not the solution for all patients with diminished MC [mental capacity] who refuse treatment. In some situations, it may do more harm than good.” Data from Denmark show that especially in patients with multiple prior treatments that were not separated by a period of good health, compulsory treatment becomes unproductive and sometimes even traumatic for the patient, increasing the likelihood of them refusing future interventions [21].

We too have observed meaningful recovery emerge even after multiple treatment courses in higher levels of care, where patient preference not to receive this care was either overridden by pressure from loved ones, their outpatient clinical team, or even by legal means including guardianship or court order. Despite this, we agree with Clausen et al. [21] that “involuntary treatment is a double-edged sword, and to further explore the ethics and efficacy of involuntary measures, future studies should also include longitudinal outcome investigations focusing on illness trajectories and recovery as well as qualitative studies on patients' and families' perspectives on involuntary treatment.” Furthermore, far from disagreeing with the potential value of such care for many patients, we only specifically identify that a narrowly-defined subcategory of patients be permitted to receive care outside standard care protocols that mandate admission to a higher level of care for certain behaviors and psychological/medical sequelae. That is, once someone with SE-AN has reached at least the age of 30 and has previously engaged in high quality care to the degree feasible, they should be supported and encouraged if they desire to be admitted to a higher level of care, but they should not be forced to do so. This decision by no means suggests that individual will progress to terminal AN,only if they subsequently further decline care that will inevitably lead to their death by malnutrition, understanding the consequences of their choice, will they meet the designation.

### Concern 3: A terminal psychiatric condition cannot be defined

Critiques recognize that death as a sequelae of AN does not necessarily categorize that illness as ‘terminal’ —any more than there might be terminal forms of depression, substance abuse, personality disorder, or any of a number of other conditions that can lead to extreme suffering and death.

### Discussion

Since terminal AN clearly involves deteriorating physiological status due to malnutrition well known to lead to death [22], this quality alone differentiates it from other psychiatric conditions. For example, literature concerning starvation describes only a few cases of survival in patients with BMI < 9 kg/m^2^ [23]. This does not imply one must have a BMI this low to die from AN or indeed that a critically underweight individual in expert care might not survive, but rather that, physiologically, when a person with serious medical malnutrition declines to take in sufficient calories to sustain their body, they are almost certain ultimately to die of malnutrition. Indeed, highly regarded eating disorder authorities now consider AN to be a “metabolo-psychiatric condition,” a claim not generally made for the other psychiatric disorders listed [24]. No one would argue that metabolic disorders can’t progress to terminal phases.

A second issue worries that defining terminal AN conflicts with treatment goals and will lead to unjustified deaths for a treatable condition.

#### Discussion

To the contrary, delineating terminal AN might equally encourage patients to discuss their concerns and feelings about futility and terminality with their clinicians, concerns that they may previously have hidden from their clinicians. Indeed, where patients with SE-AN frequently experience the distortion of not being “sick enough” to merit behavior change or optimal care, it can be validating and recovery-motivating for a patient to hear the specifics of how if they do not make changes, they will indeed die. Having respectful, thoughtful conversations over time with a trusted expert clinician about struggles, victories, disappointments, quality of life, goals, and values can allow the patient to reclaim ownership of the recovery, where the clinician acknowledges there will be support but no pressure to retry a higher level of care despite meaningful medical risk. It is not uncommon for even the most exhausted patient in this context to examine their own autonomy and choose to keep working for a meaningful recovery. Conversely, some patients might learn about the idea of “terminal AN” and attend only to the first three criteria (ignoring the fourth that involves the patient proactively choosing to stop fighting). They might ask a trusted clinician if the existence of such a concept means that they are doomed. We would posit that the instinct to process such a reaction—and even to rebel against the concept of terminal anorexia—represents a clinically meaningful opportunity to reengage with a more vibrant recovery process.

We anticipate that acceptance of the idea that terminal AN exists will be higher among certain groups of clinicians: those who have had extensive direct, ongoing, personal, frequent, prolonged bedside experience with patients who refuse more active treatments at this stage of their disorder. Some clinicians might publicly voice denial that terminal AN exists while privately acknowledging that terminality exists for some patients. Some may be influenced by a host of cognitive and affective biases and/or concerns regarding social or professional approbation [25–27], which might lead some clinicians to forms of denial, in which they essentially avoid facing the realities of these terminal states. In any case, those who persist in denying even the possibility that the condition of terminal AN exists do these patients and their families a great disservice. They would deny these patients and their families the options of more dignified deaths compared to alternatives such as the suffering and anguish of prolonged dying from complications of inanition or from stigmatized and shaming suicide.

### Concern 4: The proposed definition is not specific enough

Central to this criticism is the concern that the definition is overly broad. This criticism notes that people over the age of 30 can recover, that access to high-quality anorexia care is highly variable and imprecisely defined, and that a lack of objective data leads to unclear prognostication.

#### Discussion

This criticism crucially ignores the fourth criterion laid out in the original article. We agree of course that the definition would be overly broad if it were limited to diagnosis of AN, age over 30, and prior receipt of high-quality care. Many patients meet these criteria and do beautifully, going on to full recovery or a satisfactory life on a harm reduction plan. This is precisely why the fourth criterion exists, that the person with decision making capacity additionally identifies clearly and consistently over time that they truly decline further treatment at a higher level of care, and having failed attempts at harm reduction and palliation, accept that death will occur should eating disorder behaviors not change. Vitally, such a decision must be made under the care of highly expert eating disorders providers, because they are the most likely to be able to offer seemingly hopeless patients the strategies that may indeed permit reengagement with recovery efforts. Nor can this decision be made at a single point in time, but rather must be explored thoroughly with meaningful family engagement over time. The reality is that very few patients, even the ones who are exhausted, depleted, suffering, and unsure of what the future might hold, actually arrive at and remain consistently over time in this space. When they do, tenets of autonomy and right to choose for one’s body must prevail.

Riddle et al. correctly note that substantial barriers often exist to accessing high quality eating disorder care, including cost and insurance barriers, geographic restrictions, and personal hardships. They conclude that lack of access to resources may mean patients don’t have the opportunities they deserve to engage in exhaustive recovery efforts. To be sure, ethical principles of justice compel us to strive for equitable distribution of responsibilities, assets, and rights across a population. In that light, tying definitions of terminal AN to access to specific types of treatment for specific lengths of time disproportionately burdens patients with fewer means who may not have the resources to seek continuous expert-level eating disorder care for years or decades. Defining criteria for terminal AN is meant to cue practitioners about the potential need for a palliative approach or comprehensive end-of-life care plan. Restricting that definition only to patients who have the means to endure specific types of extensive and expensive treatment is an injustice. For that reason, the proposed criteria require but do not presuppose the type or duration of high-quality eating disorder care to protect disadvantaged populations from the burden of diminished access to end-of-life care resources.

This diagnostic subset of patients with terminal AN is not merely academic or theoretical in nature. In debating the specificity of the terminal designation, it is important to pause and consider how narrowing the definition further would play out in actual clinical practice. If one were to raise the minimum recommended age to 35 or 40 for instance, one must consider the care of a patient who is 31 years old and meets all the other criteria, has had a relationship with an expert provider for a year, and refuses further treatment and has progressive medical instability. Every attempt at harm reduction has failed because the patient cannot bear the suffering anymore, and they cannot make behavioral change that mitigates either their suffering or their medical risk. The team and family members have done everything possible to encourage alternative treatments like ketamine or deep brain stimulation, and either there aren’t resources or there isn’t willingness to undergo these. One can take the ideologically pure stance and say that this patient simply cannot be designated terminal, with no rights to access compassionate end of life care. But there is little practical recourse for enforcing this short of the parents/partner choosing to force the individual out of the house and leave them homeless unless they comply with retrying treatment. Or sedating the individual, restraining them, putting them on a plane to one of the few US states that actually has the capacity to hold and treat a such a patient fully against their will, via locked wards and intensive nursing efforts that involve court orders, involuntary medications, and forced tube feeding. This essentially impossible scenario is made even more unlikely when we take into account that only individuals with the capacity to pay for such care (via insurance or private pay) could actually access it. It is well known that in the very limited settings where such care is available, court orders regarding grave disability and mandated treatment are dropped once the payor source stops paying, even if the clinical presentation has not changed.

This practical consideration applies as well to considering whether the definition of “prior persistent engagement in high-quality, multidisciplinary eating disorder care” needs to be made more explicit and with a higher standard than outlined in the original manuscript. It would be optimal if this could be defined as recent admission to a highly expert residential eating disorder program, full weight restoration, and step-down through each lower level of care with supervised practice of life skills and established maintenance of ability to sustain appropriate eating behaviors, ongoing weight stability, and practice of alternative coping mechanisms while optimizing psychiatric medication. In fact, we strongly encourage this completeness of care wherever possible. Should we, however, establish this as the rigid minimum standard below which no one can qualify for end-of-life care?

Some providers would say yes, and many of them work within higher levels of care in the United States, where this care is more available than elsewhere in the world, and where they are accustomed to being able to control everything about a patient’s care while seeing the benefit that does accrue to many in these settings. However, the reality of outpatient care is different, as the outpatient provider controls almost nothing and relies on relationship with the patient, communication with a strong multi-disciplinary team, and engagement with the family to try and support change over time. One must also not discount that the very fact of a highly sensitive patient’s intolerance to higher level of care settings and refusal or inability to persist in such treatment—not in the early years of an eating disorder but after age 30—is in fact salient to their experience of the world and to their suffering. It is too reductionist for family or team to insist that such a patient “just” agree to go to treatment and complete a full course, presuming that this will fix everything. A patient who finds group care settings (in which they are surrounded by other ill and suffering individuals, away from home and safety) to be intolerable, even if this might prove lifesaving, must elicit our compassion and validation as providers, not our ire and frustration.

### Concern 5: Capacity considerations in the criteria do not account for the psycho-cognitive impairments in anorexia nervosa

This concern argues that decision making capacity in patients with AN regarding their treatment is impaired, and therefore they are incapable of making decisions limiting the scope of their care. Capacity to consent to or refuse a treatment requires understanding risks and benefits and weighing the pros and cons of proposed options, appreciating how they apply to one’s own condition and making and communicating a reasoned, consistent choice. These critics may argue that standardized tests of competence fail to identify many individuals with AN whose capacity is impaired.

#### Discussion

We and others (e.g. [28, 29]), take issue with the sweeping assertion that patients with AN lack capacity for medical decision-making. In the oft-cited study of mental capacity in patients with AN conducted by Elzakkers et al. [30], in which 70 adult women with AN were studied (average age 27.3, average duration of illness 8.6 years), the authors report, “Based on clinical judgment, 46 patients had full mental capacity and 24 diminished mental capacity. Based on the MacCAT-T, 43 patients had full mental capacity and 25 diminished capacity.” This is quite far from asserting that all patients with AN have diminished capacity; it acknowledges that a significant percentage of patients with severe AN retain mental capacity. By contrast, the speculative, theoretical paper of van Elburg et al. [20] implying that patterns of emotional arousal in patients with AN adds to their impaired decision-making, unfortunately contains no additional data. Although the obsessional ruminations of individuals with AN can be perplexing, clinicians should not regard the presence of body distortions and food fears as proof that these patients are unable to understand personal options and make reasoned health care decisions. Even the presence of other comorbid psychiatric conditions does not preclude decisional capacity for medical decisions [31, 32]. With respect to decision-making capacity, four traditional criteria are usually applied: understanding, appreciation, ability to reason, and communication of decision [33]. While much has been made of the disease-associated neurobehavioral adaptations in AN and their cognitive impact, those changes do not disqualify patients from having capacity de facto unless they demonstrate limitations in the above criteria. Prior meta-analyses on the topic find that most patients with mental illness, including bipolar disorder or schizophrenia, are able to make rational decisions regarding their healthcare [31]. A formal assessment of decision-making capacity by a psychiatrist independent of the treatment team may help ameliorate family member fears that such an important decision is being made in an appropriate and ethical manner, especially when AN fears and distortions can seem so irrational. An individual who wavers in their conviction or expresses different goals to different people is not yet ready to receive the appellation of terminal AN.

Additionally, to question a patient’s capacity when they are making decisions discordant with a practitioner’s values, but to accept their capacity when they are making concordant decisions, is a type of culture bias. If a practitioner believes that a patient has the capacity to elect between different types of disease-directed approaches, it would be a logical strain to suggest they do not have the capacity to elect a palliative-only approach. Careful determination of decisional capacity is required in each case [34]. We agree that a robust capacity assessment that takes into account potential neurocognitive alterations in patients with eating disorders is a valuable resource for the application of these criteria, and we encourage both the development and promulgation of an acceptable tool.

## Conclusion

Outside of the published criticisms of our original article, various groups have expressed a concern that the existence of a term like “terminal AN” could be used to deny ongoing care to an individual whose eating disorder severity has resulted in intense resistance in treatment settings or recurrent admissions without obvious benefit. However, unless the patient otherwise meets all the criteria however, including that vital criterion 4 regarding their personal choice not to continue treatment while knowing death may follow, the term cannot be misused.

Given the vital importance of this term being applied strictly and rigorously, we wish to identify explicitly that terminal AN applies only to a rare subpopulation of all those with SE-AN. Indeed, it is worth outlining the broad spectrum of patients and care goals that emphasize this point. The vast majority of individuals with AN of all ages and chronicities will fully recover, and this should always be the initial goal and be supported for any patient at any stage who wishes to achieve full recovery. Once an individual is over age 30 and has not proven able to achieve full recovery yet, *if* they feel this goal is presently out of reach, it is reasonable to work toward a satisfactory harm-reduced plan that emphasizes autonomy, relationships, and honoring the patient’s goals and values. Many live quite well in this way, and at times, the reduced pressure may yet lead down the road to full recovery.

A small subset of this group cannot find safety in harm reduction, seeming to lack a “basement floor” such that any eating disorder behavior leads swiftly to medical instability. For these individuals, a palliative care approach is appropriate, which emphasizes risk reduction, comfort, hope for improvement, and connection. Patients may live for years at this level, even as their suffering is impressive. At all these stages, those who wish to admit briefly for respite (to nourish better, halt a behavior cycle, and reduce symptoms) or longer term should be supported in but not forced to do so. It is only when the palliative approach does not work, and death becomes imminent, that the patient meets the designation for terminal AN. An even smaller subset of these individuals will live in a place where MAID is legal and will choose to obtain these medications, with yet fewer who will actually choose to take them.

To negate the concept that AN can have a terminal component is to imply that some other person, intervention, or approach would have prevented that death. This theoretical implication prevents the identification and proper care of those who are presently dying of SE-AN, do not wish to die by suicide, and do not wish to return to a higher level of care. Rather, these patients wish to receive compassionate care that validates their years-long efforts toward recovery, mitigates suffering, and honors the lives they have lived. To halt professional concern and caring at a philosophical, ideologically “pure” perspective that mandates more active treatment in practice actually judges and stigmatizes the dying. This position additionally burdens people who die of AN, compelling patients to further suffer at the end of their lives in pursuit of an increasingly impractical goal. It also places an added burden of guilt on providers and families who weren’t able to prevent their death. In our view, rather than denying the obvious, it is more ethically meritorious to realistically appreciate that some patients will die of their disease. By being able to Identify these patients, clinicians might be better positioned to reduce their suffering through palliation to improve quality of life and then for those who progress to this point, through hospice care as they approach their deaths. We submit that our proposed characteristics of terminal AN might offer the eating disorder and palliative care fields starting points for this identification, points which will clearly require refinement.

Clinical, legal, and ethical commentators in the field concur that transitions to comfort-oriented care may be appropriate when further treatment, whether voluntary of involuntary, will provide only brief improvement and is unlikely to offer sustained quality of life [35, 36]. In our view, when patients acknowledge the possibility of not being able to survive, every effort should be made to seriously consider their perspectives and offer individualized harm reduction treatment options that might make the remainder of life bearable. However, should these fail, these patients should be supported in seeking palliative care and, if indicated, hospice care. Therapeutic goals in these situations are to ameliorate suffering, protect dignity, and honor the person’s values. Families who receive such care report a marked relief when they feel permission to transition from being the law enforcement officers of the eating disorder and instead return to the most innate and natural role of a family member: to provide love and support and to bear witness.

### How can the field move forward to consider the existence of terminal anorexia nervosa and better meet the needs of patients and families struggling with this condition at the end of life?

With respect to achieving agreement on definitions and for developing formal diagnostic criteria for terminal AN (and SE-AN), the field might look to processes that resulted in the inclusion of avoidant/restrictive food intake disorder (ARFID), rumination disorder, and pica in the DSM5, propelled by the American Psychiatric Association’s Eating Disorders Work Group [37]. These efforts followed the American Psychiatric Association’s Guidelines for Making Changes to DSM-V [38] based on literature reviews and secondary data analyses that document the clinical validity of the proposed changes. Deliberations examined the quality and quantity of studies being reviewed and the strength of the evidence in each study for various measures such as prior psychiatric history, clinical outcomes, course of illness, diagnostic stability, familial aggregation and/or co-aggregation, biological markers, comorbidity patterns, treatment response, environmental risk factors, and sociodemographic factors such as age, sex, and social class, among others. In addition to diagnostic validity, these formulations include evidence of clinical utility [39].

To develop clinical guidance for managing patients with terminal AN, well developed methods based on consensus groups using Delphi methods would seem suitable. Usual stages of these processes include survey development based on literature review and core group input in which a core group develops potential consensus statements based on literature review and their own expertise, expert panel member recruitment, circulation of proposed statements to these larger groups of nominated experts, data collection and analyses for several rounds of survey votes and input, and ultimate consensus guideline development. [40, 41].

Work groups initiating these efforts could be formed via special interest groups focused on SE-AN organized through the Academy for Eating Disorders or through other organizations or mechanisms. A large pool of international scholars and clinicians in both the professional eating disorders and palliative care communities exists whose expertise could be tapped. Conceivably, these goals might coincide with the interests of family foundations, which in turn might fund the administrative support necessary to pursue these activities.

It is our hope that these efforts can result in better understanding among eating disorders and palliative-hospice care clinicians, acceptance of terminality, and improved professional practices to help patients with SE-AN at the end of life. We hope that these selected few patients may be trading a death with indignity (e.g. via suicide or in the process of a forced pursuit of aggressive care) for a much more desirable death with dignity, in which understanding and supportive family members and expert clinicians participate.

## Data Availability

Not applicable.

## Abbreviations

AN: Anorexia nervosa
SE-AN: Severe and enduring anorexia nervosa
DSM5: Diagnostic and statistical manual 5
MAID: Medical aid in dying
